# Wearable biosensors for human fatigue diagnosis: A review

**DOI:** 10.1002/btm2.10318

**Published:** 2022-05-17

**Authors:** Jingyang Zhang, Mengmeng Chen, Yuan Peng, Shuang Li, Dianpeng Han, Shuyue Ren, Kang Qin, Sen Li, Tie Han, Yu Wang, Zhixian Gao

**Affiliations:** ^1^ Tianjin Key Laboratory of Risk Assessment and Control Technology for Environment and Food Safety Institute of Environmental and Operational Medicine Tianjin P.R. China

**Keywords:** fatigue diagnosis, real‐time monitoring, sensing modules, wearable biosensors

## Abstract

Fatigue causes deleterious effects to physical and mental health of human being and may cause loss of lives. Therefore, the adverse effects of fatigue on individuals and the society are massive. With the ever‐increasing frequency of overtraining among modern military and sports personnel, timely, portable and accurate fatigue diagnosis is essential to avoid fatigue‐induced accidents. However, traditional detection methods require complex sample preparation and blood sampling processes, which cannot meet the timeliness and portability of fatigue diagnosis. With the development of flexible materials and biosensing technology, wearable biosensors have attracted increased attention to the researchers. Wearable biosensors collect biomarkers from noninvasive biofluids, such as sweat, saliva, and tears, followed by biosensing with the help of biosensing modules continuously and quantitatively. The detection signal can then be transmitted through wireless communication modules that constitute a method for real‐time understanding of abnormality. Recent developments of wearable biosensors are focused on miniaturized wearable electrochemistry and optical biosensors for metabolites detection, of which, few have exhibited satisfactory results in medical diagnosis. However, detection performance limits the wide‐range applicability of wearable fatigue diagnosis. In this article, the application of wearable biosensors in fatigue diagnosis has been discussed. In fact, exploration of the composition of different biofluids and their potential toward fatigue diagnosis have been discussed here for the very first time. Moreover, discussions regarding the current bottlenecks in wearable fatigue biosensors and the latest advancements in biochemical reaction and data communication modules have been incorporated herein. Finally, the main challenges and opportunities were discussed for wearable fatigue diagnosis in the future.

## 1 INTRODUCTION

After prolonged high‐intensity physical labor, problems, such as lack of concentration, fatigue, and suppressed immune system, are induced that causes declined immunity, damaged nervous system, and retarded alertness and coordination. This phenomenon is summarized as fatigue, which occurs more in those who perform excessive manual labor, such as soldiers, pilots, and athletes,^1^, ^2^, ^3^, ^4^ affecting individual and surroundings. According to a report by the Japanese Ministry of Health and Welfare, 59.3% of the people who died within 30–65 years of age were healthy. In terms of the cause of death, 24.3% of the total people who died from cerebrovascular disease, had headaches, and among those who died from heart failure, 29.5% reported feeling tired and burnt out. Therefore, to avoid such disaster caused by fatigue, timely fatigue diagnosis is essential for soldiers, athletes, and other groups exposed to excessive fatigue.^5^, ^6^

Biomarkers, which reflect the fatigue state of human, are broadly classified into two categories, viz. (a) physiological and (b) biochemical.^7^ The detection of physiological biomarkers, including muscle strength, heart rate, and maximum oxygen uptake, is relatively intuitive and straightforward, but the lack of quantitative standards impedes the fatigue diagnosis.^8^ However, since biochemical biomarkers, such as lactic acid, cortisol, testosterone, and others, are predominantly found in biofluids, such as blood, sweat, urine, and saliva, their detection requires collection of biofluid samples. Since, biochemical biomarkers reflect human fatigue quantitatively; biochemical biomarkers are regarded as the primary biomarker of fatigue diagnosis.^9^, ^10^, ^11^ Although traditional methods for detecting human bio‐fatigue marker, such as LC‐MS,^12^ GC–MS, and chromatography,^13^ can achieve accurate and stable detection, expensive instruments and complicated preprocessing hinder their widespread applications in real‐time practical applications.^14^ Rapid detection technologies represented by enzyme‐linked immunosorbent assay,^15^ colorimetric sensors, and test strips,^16^ are composed of biological recognition components, such as enzymes, antibodies, aptamers, and signal conversion components.^17^ Recently, biosensing methods based on colorimetric and test strips^18^ to detect cortisol and creatine kinase isoenzymes have been developed. Based on an intuitive and convenient signal reading, color charts and test strips contributed to the progress of fatigue analysis. However, these methods cannot continuously monitor the changes of fatigue biomarkers during exercise, which cannot provide timely feedback on the abnormal state of human. In conclusion, efficient, continuous, and timely fatigue analysis is still limited.^19^

With the development of artificial intelligence (AI) and advanced material science, the concept of the Internet of Everything has emerged gradually. Methods, which can eliminate the limitations of laboratory environment for real‐time detection of biomarkers are explored, and wearable sensing technology, composed of medical monitoring and wireless signal transmission, has emerged as the times require.^20^, ^21^ In this context, the advanced technology companies, such as Apple and Huawei, have already introduced integrated heart rate, pressure, and blood oxygen saturation modules into their wearable wristbands and watches for continuous monitoring of the changes in physiological states through interconnection between smartphones and detection modules. In addition, smart wearable biosensors find applications in the field of military. For example, the U.S. military has built‐in EEG sensors on military caps or headsets for conducting real‐time fatigue diagnosis of the personnels in key positions and is equipped with a fatigue warning and recovery system. Therefore, wearable biosensors have played an important role in medical diagnosis, military operations, and health care.

Wearable biosensors are biosensing devices, which can detect biomarkers accurately, instantly, and portably.^22^ Early wearable biosensors predominantly focused on detecting physiological signals, such as blood pressure, steps, heart rate, and sweating rate, which could not reflect the abnormal human state changes accurately and comprehensively.^23^, ^24^ With the development of insight of health care and disease prevention, the quantitative detection of biochemical biomarkers in biofluids has shown more possibilities.^25^ In recent years, with the development of flexible material technology and wireless communication technology, modules of wearable biosensors, including sample collection, biochemical reaction, and signal readout, are integrated in wearable biosensors, which noninvasively analyze the wearer's biofluids and output the signals in the form of electrical and optical.^26^ Then, the wearable biosensors feeds back the health data and physiological changes through wireless communication technologies, such as WiFi, Bluetooth modules, and near‐field communication (NFC).^27^ Although wearable biosensors were initially widely applied in the field of medical monitoring, but with the increasing emphasis on exercise fatigue, wearable biosensors are expected to play the vital role in military operations and sports monitoring.

As an auxiliary means of medical monitoring, wearable biosensors mainly focus on the analysis of medical diseases, such as diabetes^28^ and Parkinson^29^ for improving the efficiency of medical diagnoses and health management, and the application of wearable biosensors has been summarized and reported in detail, which proved that the wearable biosensors were suitable for medicalcare.^30^ However, advantages, such as easy portability, sweat collection, and a wide range of applications of wearable biosensors, are not fully exploited. The application of wearable biosensors in fatigue diagnosis may provide the possibility to exert their advantages fully. In the traditional diagnosis methods, the analysis of fatigue biomarkers in serum samples still occupies a significant position in fatigue diagnosis that require painful blood sampling and the larger time.^31^ Whereas wearable biosensors can fit close to body surface and therefore can stably detect fatigue in both motion and resting states with both flexibility and portability in the most instances. However, few reports focused on customized wearable biosensors for fatigue diagnosis because of the limitations in technology and lack of specific diagnosis. In the following discussion, we will discuss about applications and prospects of wearable biosensors in fatigue analysis by introducing the development in them. First, biomarkers in invasive and noninvasive biofluids were introduced, and the advantages and disadvantages of wearable biosensors in different biofluids have been explored. Thereafter, the improvement of biochemical response elements in multichannel detection, improved sensitivity, biometric recognition, and groundbreaking reports with significant impact have been reviewed. After that, the development of data modules based on different signal readout in recent years has been expounded, and their potential in continuous fatigue diagnosis has been discussed. Finally, we have outlined the existing bottlenecks and challenges of wearable biosensors in the field of fatigue diagnosis and emphasize their development prospects.

## 2 BIOFLUIDS FOR WEARABLE BIOSENSORS AND THEIR PROSPECTS IN FATIGUE DIAGNOSIS

Biological metabolism, including inorganic salts, hormones, enzymes, and biological macromolecules vary with the state of physical function.^32^, ^33^ Some biomarkers, which change significantly during exercise fatigue, are considered fatigue biomarkers. Biofluids are easy to obtain with few preprocessing steps and the presence of fatigue biomarkers are prerequisites for wearable fatigue diagnosis. Blood is a transit station for physiological metabolism and contains a rich variety of biomarkers.^34^ Compared to other biofluids, there are a wide range of biomarkers in serum with higher concentration that is an ideal sample for fatigue diagnosis. Some biosensors for the detection of fatigue biomarkers in blood were summarized in Table 1, and their sensitivity and detection performance were feasible within its applicable range. However, serum analysis is accompanied by a complicated sample collection and purification process, which requires professional technical operators. The painful blood specimen collection not only causes harm to the subjects but also causes infection and safety issues because of the pollution caused by surroundings and secreted sweat,^45^ which is not suitable for users with exercise fatigue. In addition, although some colorimetric‐based biosensing methods have been developed, strict experimental conditions are still required. To achieve user‐friendly fatigue diagnosis, fatigue analysis of noninvasive biofluids is essential.

**TABLE 1:** Biosensing strategies to detect fatigue biomarkers in blood

Detection samples target	Detection range	Output signal	Instrument dependency	References
Testosterone	5 pg/ml to 50 ng/ml	Electrochemical	Electrochemical workstation	35
Testosterone	29–290 pg/ml	SPR	BIAcore™ SPR instrument	36
Testosterone	1–1000 ng/ml	Instrumental analysis	Ultra‐performance liquid chromatography tandem mass spectrometry	12
CK‐MB	1 pM to 50 nM	Colorimetric	Smartphone and software	37
CK‐MB	0.2–625 nM	Colorimetric	Smartphone and software	38
Myoglobin	60 pg/ml to 6 mg/ml	Fluorescence	Fluorescence spectrophotometer	39
Myoglobin	1–20,000 ng/ml	Electrochemical	Electrochemical Workstation	40
Cortisol	10–500 ng/ml	Instrumental analysis	HPLC coupled to the diode array detector	41
Uric acid	1–1000 μM	Electrochemical	Electrochemical Workstation	42
Uric acid	0.1–5 mM	Fluorescence	Fluorescence spectrophotometer	43
Uric acid	0.01–1 mM	Colorimetric	Smartphone and software	44

**FIGURE 1:**
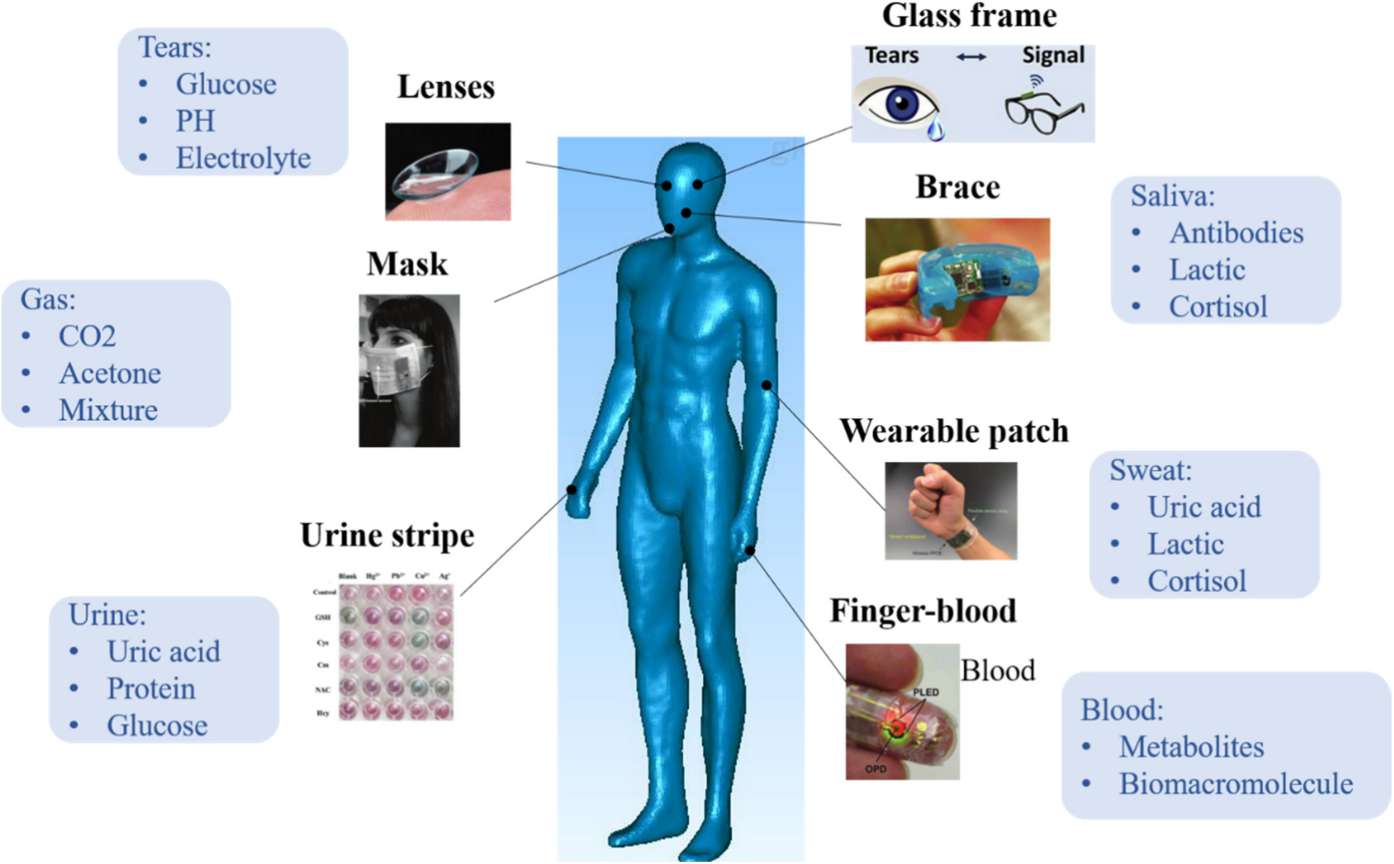
Overview of biofluid samples and their application of wearable biosensors for fatigue diagnosis

Noninvasive biofluids include tears, saliva, and sweat, where some biomarkers reflecting human fatigue are also secreted. For instance, tears are secreted by lacrimal glands and contain biological components, such as proteins, lipids, glucose, and electrolytes.^46^ In particular, the contents of biological components in tears correlate with blood and is therefore regarded as excellent biofluids for noninvasive diagnosis of local or systemic disease.^47^, ^48^ Again, saliva is secreted by the salivary glands, and the antibodies present in saliva have proven to be an effective way to diagnose HIV^49^ and intestinal infections.^50^ Sweat is secreted and excreted to the skin surface by sweat glands, which is easy to obtain noninvasively.^51^ Rich types of metabolites, such as water, electrolytes, and others, closely related to blood concentration in sweat,^52^, ^53^ reflect changes in physiological metabolism. Fatigue biomarkers, such as lactic acid, uric acid, and cortisol, are present in these noninvasive biofluid, based on which abnormal state of humans from multiple aspects can be analyzed.^54^, ^55^, ^56^ The relationship between noninvasive biofluid and blood biomarkers has been explored gradually^57^, ^58^ that is the technical basis for efficient and accurate analysis. Based on the noninvasive biofluids, which facilitates sampling, wearable biosensors can avoid painful and dangerous blood collection processes and noninvasively extract samples for analysis, friendly to wearer's daily life.^7^, ^51^, ^59^ By collecting sweat, saliva, blood, interstitial fluid (ISF), wearable glucose detection with easy operation could be realized.^60^ Except for glucose, Figure 1 shows the physiological composition of biofluids and their representative wearable biosensors. Based on the understanding of physiological metabolites in different biofluids, biosensors targeting biofluid characteristics have been developed, such as wearable masks for breathing tracking,^61^ fingertip blood sensor for measuring blood pressure,^62^ and wearable tattoos for medical monitoring.^63^ As presented in Table 2, some wearable biosensors have been developed in recent years. By collecting noninvasive biofluids, wearable biosensors can perform sensitive detection of both physiological biomarkers and biochemical biomarkers. However, deep understanding of the potential of biomarker‐rich biofluids in wearable fatigue diagnosis is still unclear. In the following sections, we will introduce advances in wearable biosensors of different biofluids and explore their potential in military operation and exercise‐induced fatigue diagnosis.

**TABLE 2:** Wearable biosensors based on different biofluids

Samples	Analyte	Biometric Elements	Monitoring mechanism	Advantages	Output signal	References
Sweat	Alcohol	Enzyme	Electrochemistry	Multitarget detection	Current	63
Sweat	Cortisol	MIT	Electrochemistry	MIP‐based on flexible substrates	Source drain current	64
Sweat	Glucose	Enzyme	Electrochemistry	Improvement of H_2_O_2_ Catalytic Efficiency	Current	65
Sweat	Cortisol	Antibody	Electrochemistry	One‐touch operation	Electrical impedance	66
Sweat	Cortisol, glucose, ascorbic acid, skin conductivity	Enzyme, antibody	Fluorescence, colorimetric	Integrated multitarget detection	Test strip, fluorescence	67
Sweat	Multiple targets	Raman signal	SERS	Detection of almost all analytes	Raman intensity	68
Sweat	Cortisol	Antibody	Electrochemistry	Wearable antibody biosensors	Electrical impedance	69
Sweat	Na^+^, K^+^	Ion electrode	Electrochemistry	Detection of electrolyte loss during exercise	Potential	70
Sweat	Glucose	Enzyme	Colorimetric	Microfluidic structure to prevent backflow	Color change	71
ISF	K^+^	Intradermal potentiometric detection	Electrochemistry	Microprobes for subcutaneous detection	Potential	72
Sweat and ISF	Glucose, lactic acid, blood pressure	Enzyme	Electrochemistry	Simultaneous detection of physiological and biochemical markers	Current	73
Sweat and ISF	Alcohol, glucose	Enzyme	Electrochemistry	Improved detection efficiency	Current	74
Skin surface	Motion, glucose	Enzyme	Electrochemistry	Monitoring sports status from multiple aspects	Current, impedance	75
Skin surface	Motion	Composite hydrogels	Electrochemistry	Monitoring external effects	Electrical impedance	76
Skin surface	Motion	Ion gel	Electrochemistry	Underwater monitoring	Electrical impedance	77
Saliva	Cholesterol	Enzyme	Electrochemistry	Specificity and stability	Current	78
Saliva	Uric acid	Enzyme	Electrochemistry	Wearable braces biosensor	Current	79
Saliva	Glucose	Enzyme	Electrochemistry	Wearable cavity sensor	Current	80
Tears	Alcohol	Enzyme	Electrochemistry	Noncontact tear detection	Current	81
Tears	Glucose	Enzyme	Electrochemistry	Wearable contact lenses for biomarkers detection	Current	82

### 2.1 Application and prospect of wearable tear biosensors

**FIGURE 2:**
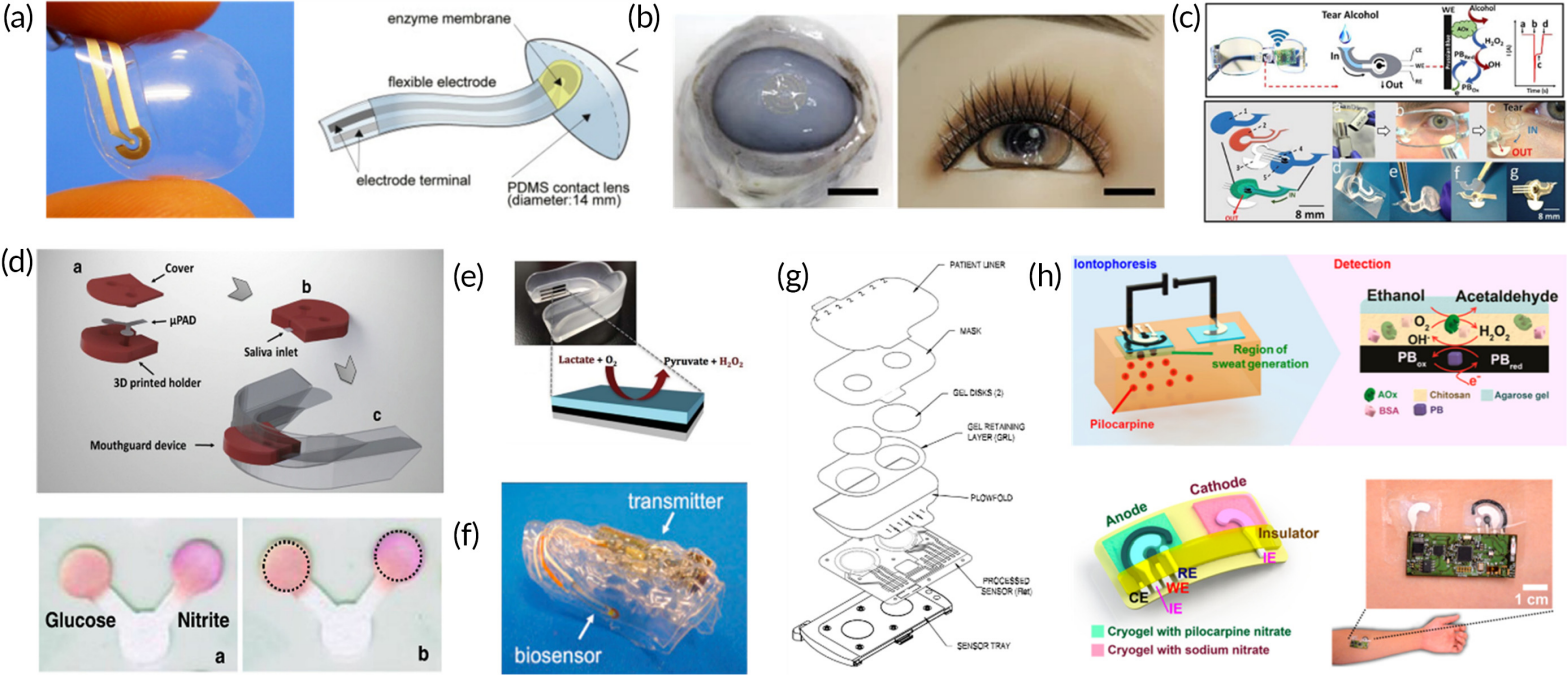
Wearable biosensors for fatigue biomarkers detection in tears, saliva, and epidermis. (a) Flexible strip‐like device for the detection of glucose in tears.^83^ (b) Wearable lenses for the detection of biomarkers closely fitting to the iris.^82^ (c) Wearable frame‐like biosensor for the detection of glucose, vitamins, and extraocular tears simultaneously.^81^ (d) The colorimetric detection of glucose based on the 3D‐printed uPADs braces.^84^ (e) Wearable lactic detection based on the screen‐printed lactic electrodes.^85^ (f) Wearable glucose detection based on the miniaturization cavitas sensor.^80^ (g) Wearable wristband for the detection of glucose extracted from the skin by iontophoresis.^86^ (h) Wearable alcohol detection based on the extraction of iontophoresis with wireless communication technology.^63^

Wearable tear biosensors have been widely developed because of the strong correlation between biomarkers in tears and blood. The original wearable tear biosensor was a flexible strip‐like device. The flexible and stretchable strip‐like device, developed by coating Polydimethylsiloxane (PDMS) onto poly(MPC‐*co*‐DMA), could detect the glucose content of tears (Figure 2a).^83^ However, the strip was difficult to fix the iris and hence was hard to adapt to daily exercise. Therefore, to improve the portability of the wearable tear biosensors, Chu et al.^87^ integrated strip‐shaped biosensor into contact lenses via preparing wearable PDMS contact lenses by fixing GOx electrodes on the flexible strip. The contact lenses could detect changes in glucose through redox reaction between glucose in the sample and glucose oxidase preloaded on the sensor, which was the first long‐time wearing biosensor. However, its wearing comfort has not improved. With the use of improved materials, such as graphene‐AgNWs hybrid materials^88^ and titanium dioxide sol–gel film,^47^ the wearable contact lens‐like biosensors^82^ could fit better in the iris and offered improved wearing comfort (Figure 2b). Despite solving the comfort issues of wearable tear sensors, attention should be given toward the possibilities eye diseases due to the heating of wireless transmission device, since contact lens‐like sensors remain in direct contact with eyes. To prevent possible eye damage from wearable tear biosensors, Sempionatto^81^ and his group designed a frame‐like wearable tear sensor, which demonstrated the possibility of detecting extraocular tears for the very first time (Figure 2c). This tear sensor was able to collect tears from the corners of eyes and could simultaneously detect glucose, vitamins, and extraocular tears via bioenzyme‐substrate reaction without any direct contact with eyeball. Currently, although the issues regarding comfort and safety have been improved, the fragility of the wearable biosensors are hinder the widespread application by people who are prone to exercise fatigue, such as soldiers and athletes.

### 2.2 Application and prospect of wearable saliva biosensors

Recently, wearable saliva biosensors have attracted researchers for real‐time monitoring of special groups of people. For instance, the wearable pacifier^89^ can adapt infant fragility and hyperactivity characteristics, providing a feasible development direction for infant physiological state monitoring without blood collection. The prototype of wearable saliva biosensors for health care was the traditional paper‐based saliva test strips, which collected saliva from subjects, followed by analyzing the responding targets with visual colorimetry or electrochemistry.^90^, ^91^ The detection of glucose and cortisol content in saliva was relatively easier by the test strips based on the colorimetric reaction. To achieve continuous monitoring, paper‐based saliva test strips were replaced by wearable 3D‐printed microfluidic paper‐based silicone braces (μPADs).^84^ Such 3D‐printed uPADs braces integrated test strip into braces, followed by fitting the oral cavity stably and detecting targets through enzyme‐substrate colorimetric reaction (Figure 2d). However, the brace required repeated removal from mouth for colorimetric analysis, which limited the widespread applicability of the braces. To improve these bottlenecks, a wearable electrochemical “brace” sensor was then developed (Figure 2e). Kim and his group^85^ integrated screen‐printed lactic electrodes into wearable saliva braces, analyzing the saliva lactic acid and outputting electrical signals continuously. With increase in lactic acid content in saliva, H_2_O_2_, by‐product of bio enzyme‐substrate reaction also increased that changed electrical signals gradually. Based on the continuous detecting‐abled wearable biosensors, wearable saliva biosensors for detecting uric acid and glucose were also developed, whose research and application results showed that the wearable saliva biosensors could adapt to strenuous exercise to a certain extent.^79^ It is worth mentioning that a further miniaturized “cavitas sensor” was later developed by Akiyoshi and his group (Figure 2f).^80^ In this work, GOx electrode was modified on the surface of polyethylene terephthalate, which could fit the teeth well and detect changes in saliva glucose (5–1000 μM). Compared to wearable tears biosensors, wearable saliva biosensors, especially the miniaturized biosensors, are more user‐friendly with better safety and sample collection efficiency, which are more advantageous in detecting the fatigue or physiological state of athletes and soldiers during their training and operation.^92^

### 2.3 Application and prospect of wearable epidermal biosensors

In addition to saliva and tears, noninvasive biofluids are also present on the skin surface. For example, sweat is vigorously secreted to the skin surface during exercise containing many biomarkers related to fatigue, and hence can be a satisfactory basis for fatigue diagnosis. What's more tempting is that wearable sweat biosensors do not need contact with mouth and eyes, thereby reducing the sensation of foreign bodies and improving comfort.^93^ Besides sweat, ISF, secreted from the superficial layer of the skin, is another biofluid with better potential in fatigue diagnosis. Unlike sweat, ISF surrounds tissue cells, directly obtaining biological metabolites diffused from the capillary endothelium without filtering,^94^ and the biological composition in ISF is reliably similar to those in blood. Recent reports have also revealed that ISF can be extracted and analyzed noninvasively by iontophoresis and ultrasonic electroosmosis.^95^ The first wearable wristband working on extracted ISF from skin surface was proposed by Tierney and his group^86^ (Figure 2g). In this work, glucose was extracted from skin by iontophoresis, and the entire module was integrated into a wristband with high accuracy and repeatability, whose relative error was considered after an appropriate interval. The wearable wristband has been commercialized and applied in a controlled clinical environment and the home environment, which contained great research value. Inspired by this work, iontophoresis was applied to promote the extraction and collection of sweat and ISF. “Wearable tattoos” coupled with iontophoresis for alcohol detection have been developed and considered as the first wearable biosensors functionalized with perspiration induced by iontophoresis (Figure 2h). The tattoos transported pilocarpine to the skin and induced sweat, where biochemical reactions occurred to produce specific detection signals. The wearable tattoo creatively constructed a wearable electrochemical biosensor that integrated drug‐loaded iontophoresis system and metabolite analysis into a single flexible patch, which solved the replacement problem between iontophoresis electrode and electrical signal analysis electrode. The design of “wearable tattoos” heralds that iontophoresis‐based wearable biosensors cannot only extract ISF but also induce sweat to increase the content of the analyzed sample. And the combined analysis of sweat and ISF was also developed based on the idea that biomarkers in two different biofluids could be detected simultaneously. In summary, wearable epidermal biosensors can analyze biomarkers of two biofluids, which is undoubtedly a huge improvement in sensing efficiency.

## 3 RESEARCH ON THE BIOREACTION MODULES OF WEARABLE BIOSENSORS

Wearable biosensors with readily available samples for analysis have made breakthroughs in recent years, but their preparation and applications in fatigue diagnosis are still not satisfactory, and some critical technical problems are still unresolved. First, various diseases are responsible for changes in biomarkers,^96^, ^97^ single channel detection of biomolecules is detrimental to the comprehensiveness and accuracy in fatigue diagnosis. Then, some biomarkers such as cortisol,^98^ creatine kinase,^99^ and myoglobin^100^ for specific fatigue diagnosis are less or absent in biofluids but blood. For detecting human fatigue state in early stages, the traditional wearable biosensors need further improvement in terms of sensitivity and ease of preparation. Finally, most of the modern biometric devices work predominantly based on bio enzyme‐substrate reaction, which are suitable to detect small biomolecules, such as glucose and lactate, while the detection of biological macromolecules is troublesome. The wearable biometric elements are required to be further improved to expand the monitoring ranges. This section will enlighten the recent development of biosensors in multichannel detection, sensitivity, and biometric elements.

### 3.1 Multichannel detection of wearable biosensors

**FIGURE 3:**
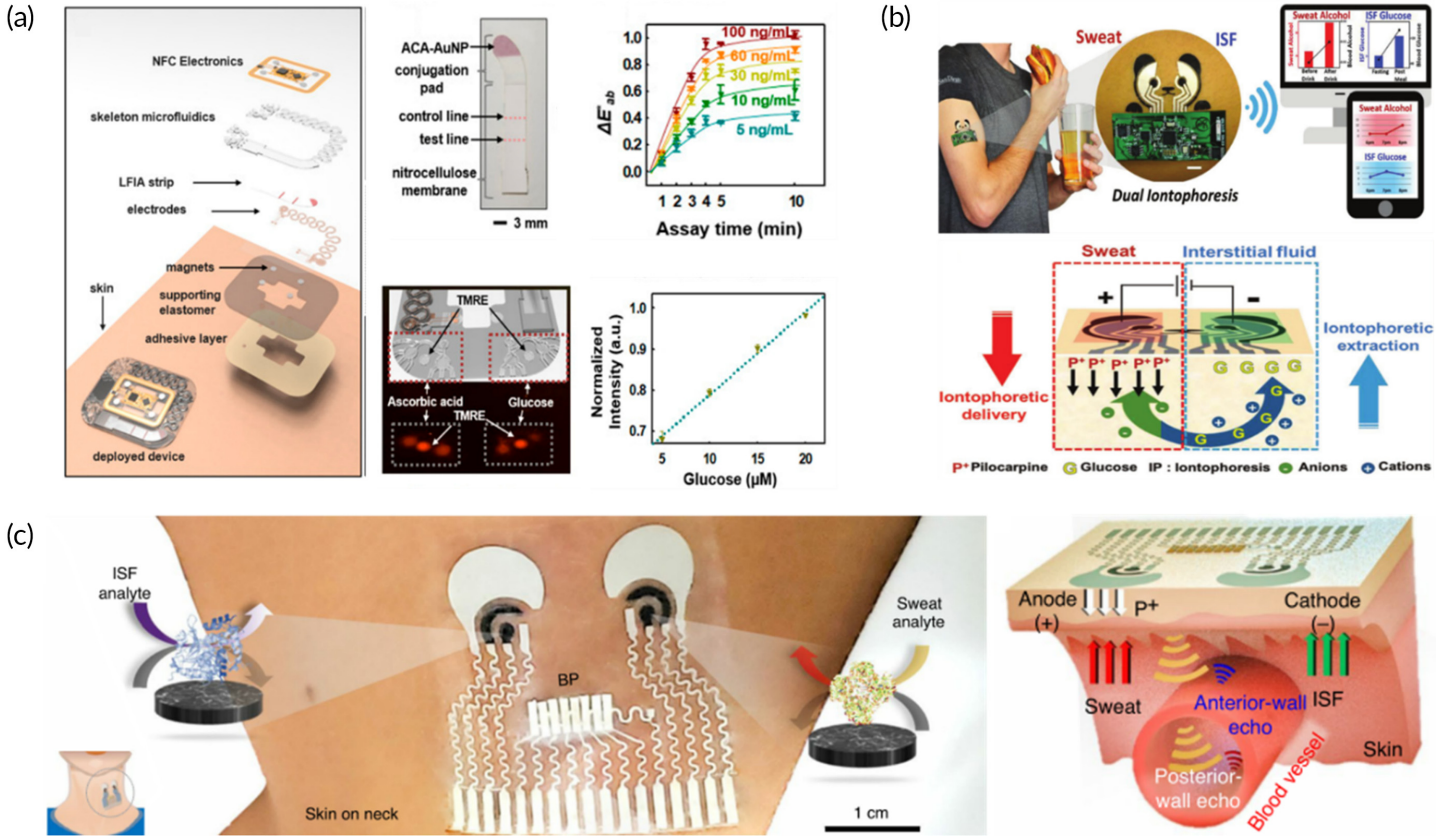
Wearable biosensors for the detection for multifunctional detection. (a) Wearable optical patch for detecting multiple biomarkers based on the combination of colorimetric stripe and fluorescent probe.^67^ (b) Wearable sweat and ISF biosensors for the detection of multi‐targets extracted from anode and cathode.^74^ (c) Wearable biosensors for the detection of multi‐biomarkers and blood pressure in a single patch.^73^

Except for fatigue, diseases, such as diabetes^96^ and gout^97^ also affect the concentration of ion and small biomolecules in noninvasive biofluids, preventing the specificity and accuracy of fatigue diagnosis. However, to improve the accuracy of fatigue diagnosis, it is essential to detect multiple biomarkers simultaneously to avoid nonspecific diagnosis. With the development of design and fabrication technology of wearable biosensors, a variety of structural designs have been applied to wearable multidetection. A classic idea for wearable multitarget design has been realized through integration of different sensing modules, based on which a fluorescent‐lateral flow colorimetric patch for cortisol, vitamin C, and glucose detection has been developed (Figure 3a). In this work, glucose and vitamin C could be detected by the enzymatic reactions between preloaded bio‐enzymes and the analytes, and the cortisol was detected by observing the detection line on the embedded lateral flow test strip. Although the integrated colorimetry and fluorescence modules have achieved multichannel detection and improved accuracy for fatigue diagnosis; yet, some limitations have been there. First, the wearable sensing modules integrated biosensors have required additional instruments, such as fluorometer, for quantitative detection. Moreover, the integration of different sensing modules has been relatively complex, and the uniformity has to be improved. Finally, the device has depended largely on sweating, which is unavailable during recovery and rest after exercise fatigue. To adapt wearable fatigue biosensors to complex training and operating environments, it is very crucial to reduce instrument dependence and improve integration. Several satisfactory technologies have been developed and provided guidelines. The classic integrated multidetection structure of wearable biosensors has been designed by Wang and his group.^74^ Herein, a wearable dual‐iontophoresis biosensor has been developed on a single flexible substrate, which has performed reverse iontophoresis and iontophoresis in cathode and anode chambers for collecting ISF and sweat, respectively (Figure 3b). Specifically, pilocarpine has been loaded on the anode electrode and repelled positively charged cations (pilocarpine) to skin, absorbing negatively charged anions from epidermis, while the opposite has been accurate at the cathode. In the process of anode–cathode convective flow, neutral molecules have also been induced to flow so that the biomarkers of different electrical properties have been extracted from cathode and anode, respectively. The innovation of device has generated and collected two noninvasive biofluids on‐demand without intense activities, and different biometric elements can be embedded separately to achieve multitarget detection without interference from each other. Based on dual‐iontophoresis system, wearable biosensors capable of detecting both physiological and biochemical signals have been developed (Figure 3c). Sempionatto and his group^73^ integrated blood pressure monitoring module into biochemical‐detection dual‐iontophoresis. Blood pressure detection has been achieved by detecting the return time of sound waves system through ultrasonic transducer. The hydrogel layer has been placed within physiological and biochemical systems to prevent acoustic and electrochemical signal interferences. The pioneering significance of this work is that it has first introduced blood pressure detection into wearable biochemical reactions, which has expanded the range of wearable detection to provide more perspective for fatigue analysis with the lower signal‐to‐noise ratio. Another wearable biosensor for multitarget detection was developed by Guozhen Liu and her group.^75^ In this work, hybrid hydrogel nanocomposites were used as flexible substrates. Based on the nanoscale hierarchical structure, good electrochemical properties and mechanical strength were performed. The hybrid hydrogel was proved to be a reliable flexible substrate that can stably monitor glucose changes and reflect external stimuli during exercise, which can contribute to the understanding of the human fatigue from multiple perspectives. Compared to multiple detection modules integrated wearable biosensors, electrochemical‐based all‐in‐one wearable biosensors have been easier to operate with a unified structure, and wireless data transmission could record the healthcare information, which has been more suitable for conditions of lack of instruments and equipment during military training and daily operations. The other advantage of these sweat‐ISF designs has been that fatigue analysis and re‐examination could be performed during rest without violent sweating, which is beneficial to protect fatigued personnel and prevent the appearance of overwork.

### 3.2 Sensitivity of wearable biosensors

**FIGURE 4:**
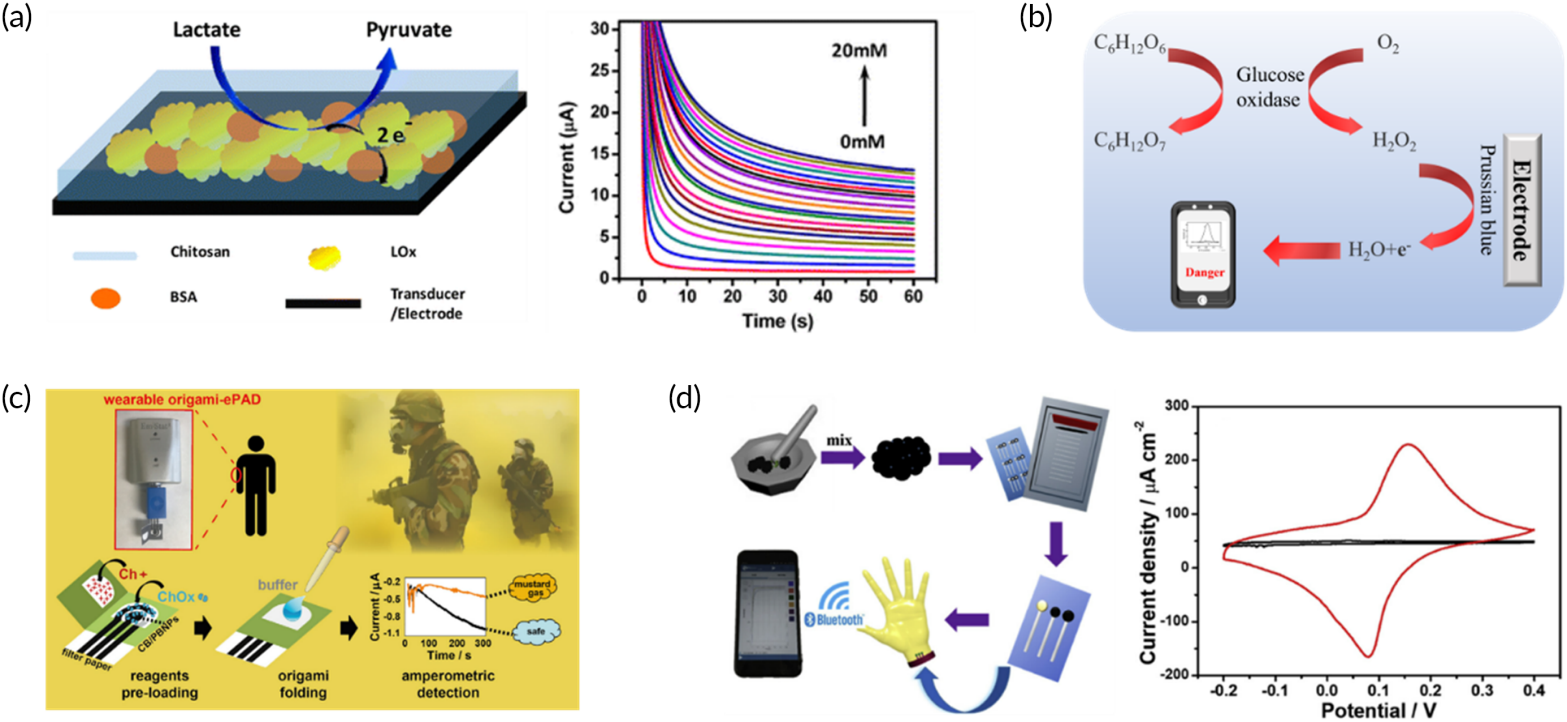
The performance improvement of the wearable biosensors in sensitivity and easy preparation. (a) Wearable biosensors for lactate detection based on the enzymes‐substrate reaction and the reduction by the redox medium.^110^ (b) Mechanism of the wearable current sensor for biochemical detection. (c) Wearable origami‐like biosensor for the detection of mustard using preloaded regents and PB‐CB nanomaterial.^111^ (d) Wearable glove for modified with functional nanocrystalline Berlin green with improved detection performance.^65^

The quantitative analysis of biomarkers that reflect physiological changes is a prerequisite for specific and accurate detection. However, some challenges still limit their development in fatigue diagnosis. On the one hand, concentration of biomolecules is lesser than that in blood because of the filtering of extracellular matrix that is important for the improved sensitivity. On the other hand, the complex preparation process of PB‐modified electrodes is also not conducive to the general development and application of wearable biosensors. Researchers have explored the solutions to the above two bottlenecks. Normally, bioselective electrodes are mainly composed of a bare electrode, a specific biological enzyme, and a redox mediator.^101^, ^102^ Common bio‐enzymes for wearable biosensors, such as glucose oxidase, lactate oxidase, and uricase,^101^, ^103^, ^104^ could carry out reversible or irreversible bio enzyme‐substrate reactions to generate by‐products, such as H_2_O_2_.^105^ Then, the electrical single was changed based on a key‐lock mechanism where H_2_O_2_ was reduced by the redox medium.^106^, ^107^ Therefore, the preparation of redox mediator electrodes is the key to improve the sensitivity and the ease of preparation. The most common redox mediator Prussian Blue (PB) was initially proposed by Neff et al.^108^ and then applied in wearable biosensors.^63^, ^109^ Wang and his group^110^ developed the wearable biosensors for real‐time detection of lactic acid in sweat in which PB was utilized as redox medium for the very first time (Figure 4a). After strenuous exercise, the anaerobic respiration was enhanced, and more lactic acid was metabolized, which then reacted with lactate oxidase and generated H_2_O_2_. Then the as‐obtained H_2_O_2_ was reduced to H_2_O and O_2_ by PB, where the electrons were transferred with changes in electrical signals. The key‐lock wearable lactic acid biosensor showed the possibility of detecting small molecules in wearable devices (Figure 4b). However, complex synthesis, poor sensitivity, and failure to detect fatigue biomarkers in a timely manner may delay the diagnosis of fatigue and cause accidents. In summary, to improve the detection performance of wearable biosensors in fatigue diagnosis, it is necessary to improve the signal conversion efficiency and simplicity of preparation. The essential requirement of the two is to improve the redox mediator.^112^

Functional nanomaterials were modified on bioenzyme electrodes to improve the sensitivity. The preparation of PB catalytic electrode constituted of the three major steps, such as drop‐casting, electrodeposition, and inkjet printing. However, this preparation method took a long time with poor repeatability and low efficiency. To improve the preparation efficiency, a PB‐CB‐modified composite electrode, composed of PB and carbon black (PB‐CB), was developed.^113^ Later Arduini and his group^111^ designed an origami‐like wearable biosensor using PB‐CB‐modified electrodes for detecting Mas in the environment with the limit of detection of 0.019 g/m^3^ in aerosol phase that proved the effectiveness of PB‐CB electrodes (Figure 4c). Herein, reagents and components were integrated on paper‐based biosensors. A ready‐to‐use wearable Mas sensor could be completed in only three steps, and PB‐CB was modified on the screen‐printed electrode (SPES) for on‐site detection. Compared to traditional PB electrodes, PB‐CB‐modified electrodes were prepared based on the typical office paper, which minimized decontamination and imparted higher sustainability. It could be prepared within 2 h and was more stable in dry condition for 3 months. However, the catalytic efficiency was still limited. Progressively, a wearable biosensor based on Fe[Fe(CN)_6_] nanomaterials was reported. The Fe[Fe(CN)_6_] was a crystalline PB‐like structure, which could be induced to prepare nanocrystalline Berlin Green (BG).^65^ In the BG electrode, a low voltage of only 50 mV was required to catalytic H_2_O_2_ that could be measured as changes in electrical signal. Low‐power consumption avoided decomposition of biomolecules in biofluids such as uric acid and ascorbic acid, improving the stability and signal‐to‐noise ratio of biosensing. The high catalytic efficiency provided good application prospects in low concentration detection. Integrated with BG, Zhu and his group designed a wearable nitrile glove with simple preparation, high efficiency, and stability (Figure 4d). Overall, wearable flexible electrodes based on advanced nanomaterials can play an excellent role in improving biosensing performance of wearable biosensors. In fact, some nanomaterials with excellent biocatalytic sensing capabilities are expected to be further expanded. For example, 2D‐MOF/Au materials, formed by modifying AuNPs on 2D‐MOF materials (metal organic framework), possess the properties of both glucose oxidase and peroxidase, to exhibit the improved biosensing efficiency.^114^ In addition, based on the gas enrichment ability of 2D‐MOF materials, sample collection rate and detection efficiency of wearable gas biosensors can be improved. These wearable gas biosensors have the potential in detecting fatigue biomarkers in breath samples and toxic gases, such as sarin and mustard in military operations.

### 3.3 Selective elements for biomacromolecules detection

**FIGURE 5:**
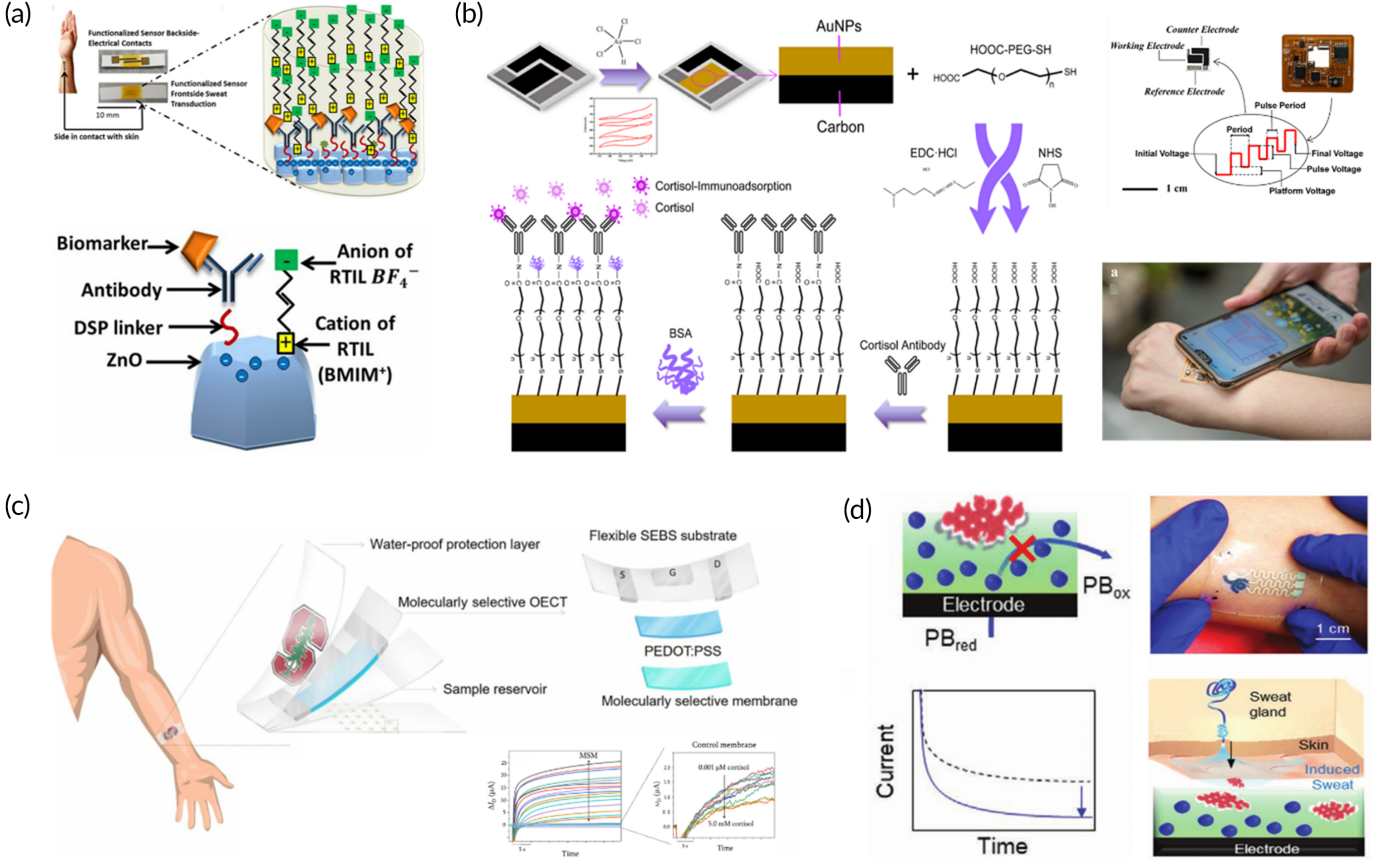
Development of Antibody and Molecular Imprinting Technology in wearable biosensors. (a) Wearable antibody biosensors for the detection of biomacromolecules.^117^ (b) A battery‐free, wireless, and flexible electrochemical patch for in situ analysis based on the antibody–antigen reaction.^118^ (c) Wearable patch based on the molecularly imprinted recognition membrane based on the current change changed before and after the cortisol was captured.^64^ (d) Electropolymerized polypyrene MIP electrode mixed with cortisol and Prussian Blue for cortisol detection.^119^

The development of multichannel detection and sensitivity of wearable biosensors provides hope for flexible fatigue diagnosis. However, the present biometric element can only detect the content of ion or small biomolecules, which cannot fully and specifically reflect human fatigue. For example, cortisol, a biomarker present in noninvasive biofluids, is increased significantly during exercise fatigue. However, traditional biological enzyme‐substrate biorecognition methods cannot detect cortisol, which poses a challenge to reliable fatigue diagnosis.^115^, ^116^ In recent years, antibodies and molecularly imprinted technologies have been implanted in wearable sensors to enable portable cortisol detection. Initial wearable cortisol biosensors were developed based on the recognition of antibodies with cortisol. Prasad and his group^117^ modified the cortisol antibody on skin‐attached test strip to detect changes in cortisol (Figure 5a). This method relies on antibody–antigen recognition for cortisol detection, but poor comfort and complex operation and signal reading process limited its application, which has been replaced later by researches. To improve the integration and portability of wearable biosensors, a battery‐free, wireless, and flexible wearable patch was then reported for cortisol detection^118^ (Figure 5b), in which device electrochemical detection modules, such as differential pulse voltammetry (DPV) and NFC, have been integrated on the flexible substrate. The DPV module has been used to detect changes in electric signal before and after antibody binding to cortisol, whereas the NFC module has been used for stable power supply and signal collection. Compared to the wearable antibodies strips, the battery‐free, wireless, and flexible wearable patch has been more convenient and comfortable because of the skin‐friendly, flexible, and extensible substrate. The NFC module could reduce unnecessary operations, realizing wireless data transmission, and collection. With the maturity of wearable antibody‐sensing technology, it provides certain support for wearable fatigue diagnosis. However, the poor stability of antibodies may make them susceptible to the interference present in the environment, such as ion concentration, pH, and other substances.^120^, ^121^ Thus, stability and timeliness of antibody‐based wearable biosensors still need further monitoring and verification.

In addition to antibodies, a groundbreaking research was reported, where the molecular imprinting technology (MTT) was integrated into the wearable biosensors for stable and reliable detection of cortisol. MIT refers to a biometric element, where the detection target is rooted in functional polymers, forming molecular templates with molecular imprinting. After the molecular templates are eluted, molecularly imprinted polymers with “molecular memory” are formed that can specifically bind with bio‐analyte.^122^, ^123^ Based on MIT, Salleo and his group^64^ developed the first wearable molecularly imprinted recognition membrane (MIRM) with “cortisol imprinting,” followed by installation within sweat collection and signal transduction layers (Figure 5c). Changes in cortisol were monitored by changes in current. When cortisol in sweat was explicitly recognized and captured by MIRM, the membrane hole was sealed, reducing the ion transporting and flowing rate. The source‐drain current (ISD) upon gating the channel was then changed. Similarly, Wang and his group^119^ developed a nonpressure cortisol sensing platform, which accurately detected cortisol in human sweat via one‐step, fast, reproducible, and high sensitivity cortisol sensing (Figure 5d). Herein, cortisol and PB were mixed as template and embedded redox probe, respectively, synthesizing the electropolymerized polypyrene (PPY) MIP electrode for cortisol detection without labels or external oxidative reduction probes. Unlike Salleo's design, in this work, the application of PB probes improved detection sensitivity, and the collection layer of biofluids consisted of a highly porous, permeable, and sweat‐absorbent polyvinyl alcohol (PVA) hydrogel. The PVA hydrogel exhibited the excellent permeability and lower impedance, which was easy to collect natural sweat. The MIP‐based wearable biosensors were more stable and robust to the influence of interference with easy preparation steps, which became popular in wearable biosensors. In terms of diagnosing fatigue of military soldiers and athletes, MIP‐based wearable biosensors can adapt to relatively complex environments.

**FIGURE 6:**
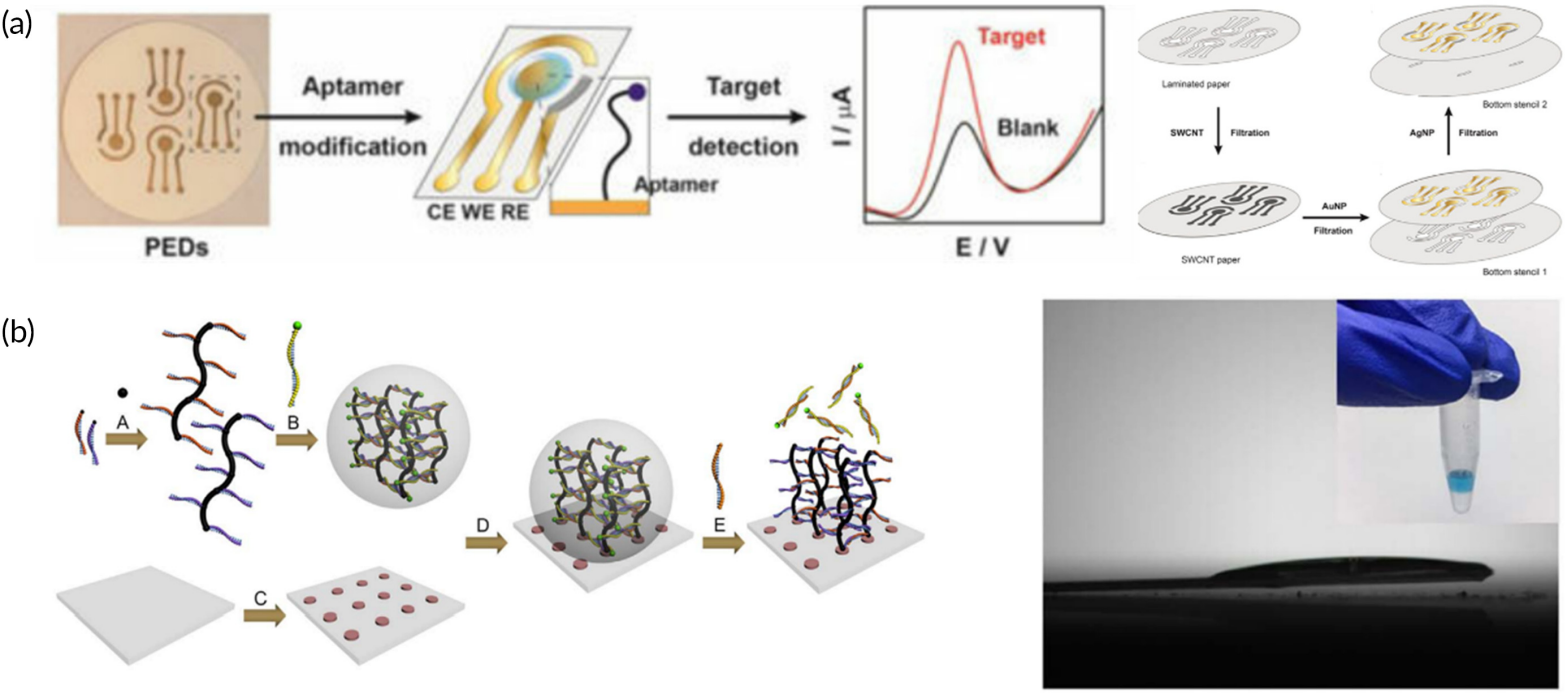
Development of DNA biometric elements in wearable biosensors. (a) The paper‐based aptamer‐modified electrochemistry biosensor fixed with different nanoparticles.^126^ (b) Paper‐based electrochemical biosensors loaded with DNA hydrogels.^127^

Another common biometric element is the aptamer. The aptamer is an oligonucleotide sequence obtained by systematic evolution of ligands by exponential enrichment (SELEX) and form a specific secondary structure after recognizing the target.^124^ Based on the advantages of low cost and stability, aptamers were widely applied in toxin and biomarker detection.^125^ However, because of the complicated modification process on flexible substrates, aptamer‐modified electrodes was yet to be developed. The simplest method was to modify the aptamer generating easy‐to‐prepare paper‐based aptamer biosensors (Figure 6a). In this work, aptamer modified with redox label MB was fixed on the three‐electrode paper‐based substrate. After recognizing cortisol, the structure of aptamer changed, shortening the distance from the surface of electrode to redox label MB. Then the electrical signal changed because of the difference in distance between redox label MB and electrodes.^126^ Another development of aptamer biosensors is to construct flexible DNA hydrogels with aptamer‐target recognition ability. Ding and his group^127^ modified DNA hydrogel which could recognize miRNA on the indium tin oxide electrode, and the change of electrical signal was then detected through dissociation and formation of DNA hydrogel (Figure 6b). Compared to general aptamer technologies, DNA hydrogels are softer and easier to integrate into wearable biosensors. However, most of the biosensing methods based on DNA aptamers and DNA hydrogels are limited to paper‐based biosensing stripes, and their stability in practical wearable biosensors require studies.

In summary, antibodies, aptamers, and MIT were primary wearable biometric elements. All of them could specifically detect biomarkers in noninvasive biofluids but also differs from each other. Due to the stability and reusability of MIT, wearable MIT biosensors have attracted the researchers. However, the multitarget detection of wearable biosensors still needs to be further developed. Wearable antibodies and aptamers biosensors have good prospects in multitarget detection, but their stability on flexible substrates is required to be improved. To further improve the efficiency and stability of wearable fatigue diagnosis, it is necessary to optimize and develop new biometric elements to solve the above bottlenecks.

## 4 SIGNAL MODULES FOR WEARABLE BIOSENSORS

To continuously detect the concentration of secreted fatigue biomarkers during exercise, rapid response and signal readout are essential. The signal modules of wearable biosensors can be mainly divided into two types: optical signal and electrical signal. In the first two sessions, we briefly discussed the development of sample collection and biochemical reaction modules of wearable biosensors. The following discussion will summarize the advanced signal readout modules along with their prospects and challenges in wearable fatigue diagnosis.

### 4.1 Optical signal module

**FIGURE 7:**
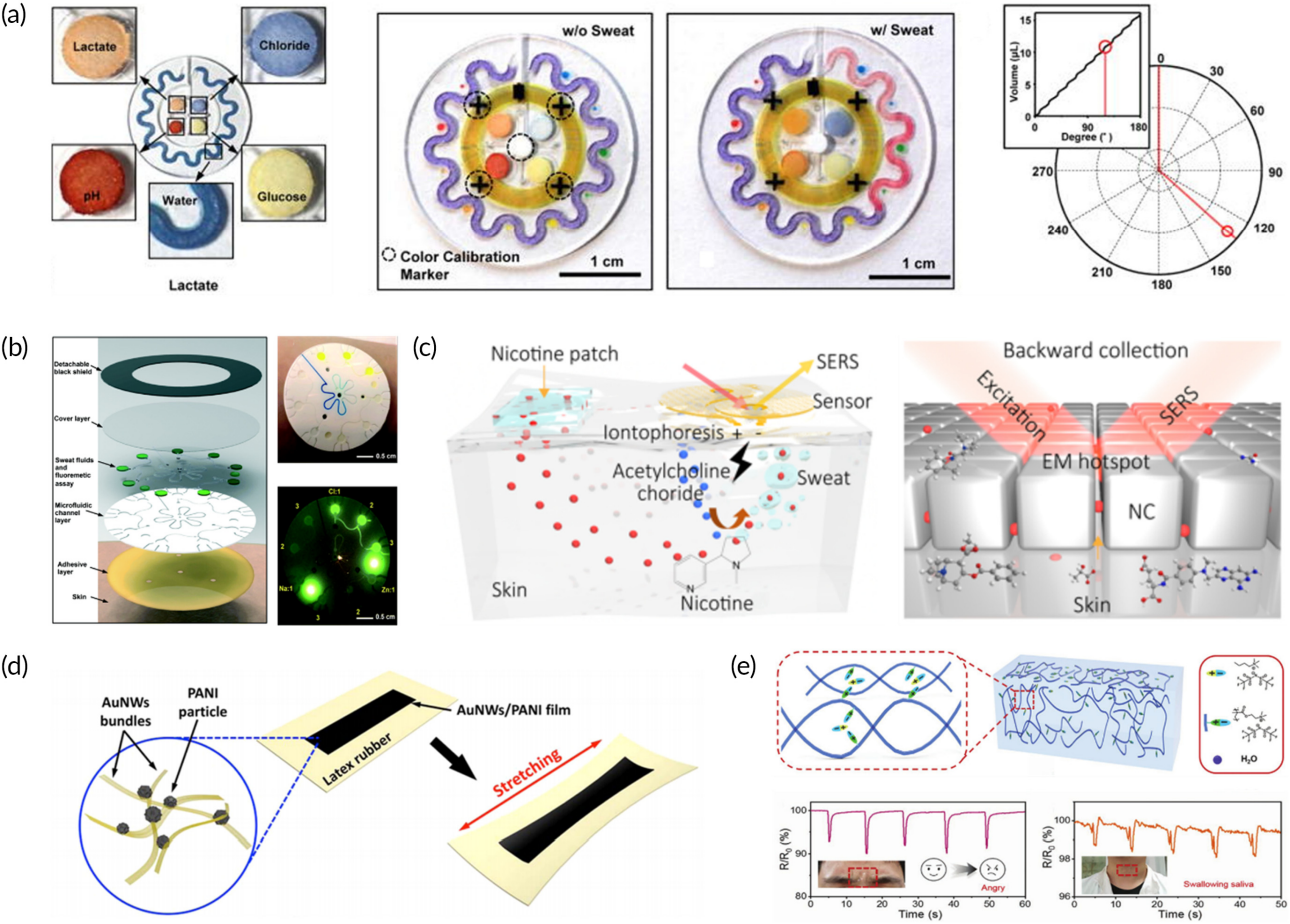
Signal output element of wearable biosensor. (a) Wearable colorimetric biosensor monitors the real‐time changes of multiple markers, including lactate, glucose, pH, chloride, and sweat flow rate based on the pioneering microfluidic system and integrated NFC module.^131^ (b) Wearable fluorescence biosensor for detecting Na + and Zn^2+^ in sweat with a fluorescent probe attached to the flexible substrate.^132^ (c) Wearable Raman biosensor with the “universal” detection of biomolecules based on the enhanced fingerprints on the flexible substrate.^68^ (d) Wearable tattoos with better elasticity and extensibility based on AuNW films.^133^ (e) Fluoride‐containing hydrogel for detecting changes in human muscles and water environment.^77^

Optical biosensors convert biochemical reactions into optical signals, including colorimetric, fluorescent, and Raman signals.^128^, ^129^, ^130^ The wearable colorimetric biosensor designed by Rogers and his group^131^ was considered to be the pioneering research on wearable optical biosensors (Figure 7a). Herein, a microfluidic system was designed for sweat extraction and collection, in which the color changed according to the specific biological reactions in microfluidic channel via monitoring changes in multiple markers, such as lactate, glucose, pH chloride, and sweat flow rate. The chromaticity data in the channel could be transferred to the integrated NFC module and quantified by software. The device did not require power supply equipment, which undoubtedly contributed to miniaturization and improved comfort of wearing.^134^, ^135^ The other advantage was that optical signals, especially the color changes,^132^, ^136^ were more intuitive and visible in absence of equipment, and the gray value of reaction solution could be analyzed by ImageJ and smartphones.^137^ Many colorimetric wearable biosensors have been gradually reported for reference and improvement, such as superabsorbent polymer valves for continuously analyzing chloride concentration^138^ and multichamber function valves for cortisol detection, that have facilitated the application of wearable biosensors.

The latest advancement in wearable optical biosensors has been accomplished by incorporating fluorescent and Raman probes into the skin‐interfaced system. The first wearable fluorescence biosensor attached a fluorescent probe to the flexible substrate and analyzed concentration of Na^+^ and Zn^2+^ in sweat (Figure 7b).^66^ Here, the microchannel network array and prefilled microreservoir were designed to collect sweat, and fluorescent probes could react with the target, achieving quantitative and rapid analysis. The fluorescence patch proved the versatility of fluorescent‐based wearable biosensors and capability of multichannel detection. In addition, colorimetric‐fluorescence wearable biosensors have also been developed based on the lateral flow stripes and fluorescent probes^67^ (Figure 3a). Another optical signal‐based wearable plasmonic‐metasurface sensor has been developed by Ying and his group.^68^ The wearable plasmonic‐metasurface sensor has been based on surface‐enhanced Raman scattering (SERS), which has almost realized the “universal” detection of molecular fingerprint. Each molecular own its specific “molecular fingerprint,” which could be faintly observed by Raman spectroscopy. With the help of gaps between rough‐surfaced nanoparticles or metal nanoparticles close to each other, the “molecular fingerprint” could be highlighted by enhancing the Raman scattering signal in hot spots. Ying and his group have introduced SERS gaps (hot spots) into wearable biosensors, where a flexible plasma substrate with SERS activity has been embedded, achieving universal molecular detection through the recognition of “molecular fingerprints” for the very first time (Figure 7c). In theory, the “fingerprint”‐based wearable biosensor could resist interference caused by integrated modules and diagnose fatigue comprehensively and specifically. However, the repeatability and continuous monitoring capabilities are still required to be studied, and the preservation and structural stability of the “hot spots” on the plasma surface in daily use are also another challenges in practical application. Optimized materials and structure design may overcome some of the problems.

### 4.2 Electrochemical signal module

Unlike optical biosensors, electrochemical biosensors output biological reactions as electrical signals. Typically, a pair of conductive silver/silver chloride electrodes are integrated into flexible three‐electrode system to prepare wearable “tattoos” and “patches” based on screen printing technology. Then, wireless transmission devices, such as WiFi,^139^ Bluetooth,^27^ and NFC module,^140^ are integrated and transmit signals to the terminals (e.g., laptops, computers, and mobile phones) for continuous tracking and monitoring changes in electrical signals. According to the principle of signal change, the electrochemical response signal includes current, potential, and electrical impedance. Although wearable electrochemical biosensors require additional power supply devices, their data can be detected through sensitive electrochemical signal changes without additional equipment. With the development of portable workstations, wearable electrochemical biosensors have shown great potential in continuous and stable fatigue monitoring. The pioneering breakthrough in wearable electrochemistry biosensors was proposed by Wang et al.,^133^ in which, wearable electrochemical tattoos with better elasticity and extensibility have been designed based on screen‐printing technology and silicon‐based substrate. Polyaniline particles have been doped into gold nanowire (AuNW) films, leading to 10 times enhancement in conductivity and about eight times improvement in sensitivity (Figure 7d). Besides, by integrating the wireless circuits, the better electrical conductivity and sensitivity have been utilized. The polyaniline particles AuNW films based materials with outstanding water resistance and durability have promoted the development of flexible substrates. Inspired by this research, more advanced materials, such as gel^141^and PDMS,^142^ have been reported for preparing wearable biosensors of improved comfort and detection performance. It is worth mentioning that the ionized hydrogel developed by Wu and his group^77^ can be applied for detecting underwater environments (Figure 4b). The ionized hydrogel was based on ion–dipole and ion–ion interactions between ionic liquids. Because of the fluorine‐containing ionic liquid introduced during synthesis, the hydrogels were hard to bind water, thereby forming strong adsorption and self‐healing hydrogels. The wearable hydrogel device accurately sensed human and underwater environment changes based on the difference in electrical impedance of number of fingers and shape of muscle. Based on the signal changes, a cryptographic mechanism, which achieved underwater communication, was established. This innovation indicated that wearable biosensors can not only monitor the changes of biomolecules, but the detection of physiological changes and instant messaging can also be realized, which contains great military significance and flexibility in movement monitoring. In addition to the monitoring of physiological changes, wearable biosensors, which monitored changes in biomarkers based on electrical impedance have also been developed. Lee et al.^66^ designed a wearable impedance biosensor for simple and passive sample collection (Figure 7e). Here, sweat was passively absorbed and transported through a microfluidic device, followed by opening transport valve through the wearer's pressing. The biochemical reaction (i.e., antibody–antigen reaction) occurred in reacting chamber after maxing of the sample via performing signal change of electrical impedance. With the design of distinguished functional chambers, the biochemical reaction could be carried out without mutual interference, which improved reusability and portability of the device. Another widely used electrochemical signal module is the current. Wearable current biosensors can monitor changes caused by biochemical reactions in real time, which have been applied in glucose^74^ and lactic acid detection.^143^ The first wearable current biosensor was used for glucose detection.^86^ When the biologically recognizable element specifically recognized the substrate, GOx reacted with glucose to generate current change in reaction chamber. The signal could be read through wireless transmission equipment. Wearable biosensors, based on current monitoring can also monitor changes in multiple samples simultaneously by iontophoresis. The classic wearable current biosensors of single‐target and multitarget detection were developed by Joseph Wang and his group, which have been explained before.^63^, ^74^ In conclusion, although wearable electrochemical biosensors require an external power supply, they perform well in monitoring efficiency and practical applications.

## 5 CONCLUSIONS AND PERSPECTIVES

Here, the latest advances of wearable biosensors and their application potential in fatigue diagnosis have been summarized. First, classical wearable biosensors for the detection of various biofluids and their application potential toward fatigue diagnosis have been reviewed. Emphatically, to diagnose the fatigue accurately and conveniently, saliva, sweat, and ISF seemed to be better substrates. Thereafter, the focus has been shifted to the core of wearable biosensors and improvement of the biochemical reaction module in multitarget detection, sensitivity, and biometric recognition ability. The development of biochemical reaction modules and their contribution toward fatigue diagnosis have been discussed. Finally, the pioneer researches on the signal output modules have been briefly described, along with the improvement of fatigue diagnosis efficiency of the wearable biosensors based on the SERS flexible substrates and electrical impedance signal. Unlike orthodox biosensors for fatigue diagnosis, the new generation wearable biosensors can detect the changes in biomarkers and diagnose fatigue noninvasively and rapidly. All the progress benefitted from the development of optimized structure designs, advanced materials, and wireless communication modules. With the development of various biosensing and advanced material technologies, wearable biosensors could fit the skin more closely with little impact on daily life, and data could be read in limited on‐site testing. Wearable biosensors offered a method to detect biomarkers rapidly and directly, which just met the needs of accuracy and timeliness of fatigue diagnosis. The widespread applications of wearable biosensors can prevent physical workers from being overworked and improve their training efficiency.

Although wearable biosensors have made significant progress in continuous health monitoring, some challenges are still faced. A fundamental problem is the power supply system for biosensors. For wearable electrochemical biosensors, power supply system is the precondition to monitor changes in electrical signals and induce iontophoresis. In addition, almost all wearable biosensors for continuous monitoring require power supply for wireless communication devices. Therefore, though power supply system is very important, yet is often overlooked for wearable biosensors. As a component in direct contact with human body, the power supply system must be flexible, stretchable, and fit well with skin and mouth, so that the device should not fall off or be uncomfortable during exercise. Currently, two resolution directions are desirable. One approach is to combine power supply systems with wireless transmission systems, such as combining power sources with NFC modules, thereby reducing size of wearable biosensors via maintaining, monitoring, and warning capabilities. Although a few flexible and skin‐friendly embedded power‐NFC substrates have been reported, high‐power consumption has implied that the long‐term applications of wearable biosensors need further investigations. The other promising way to improve is by incorporating self‐powered fabrics. Typical self‐powered systems convert bioenergy^144^, ^145^ into electric energy based on triboelectric nanogenerators (TENGs), and the as‐collected electrostatic energy can further be conducted to flexible substrates. Recently, TENGs have been integrated in shoe‐soles and cloths to generate walking energy, and a self‐powered and self‐sensing energy textile has been developed having satisfactory power efficiency. Another major problem is the restriction of capabilities of wearable biological monitoring. Although wearable multichannel biosensors, able to monitor changes simultaneously in the plurality of biomarkers have been reported, the monitoring range is still limited. Because of the poor compatibility of biologically recognizing elements, integration of biological enzymes, MIP, and antibodies, and physiological modules is difficult to implement that limits the understanding of physiological function from multiple aspects. Some of the biosensors, which assembled these biometric elements, required integration of various modules and different signal outputs, which are troublesome in terms of preparation and signal acquiring. Finally, the surroundings interference and accuracy of wearable biosensors during fatigue diagnosis are still unexplored. Although there are many challenges, with the development of advanced materials and the increasing importance of fatigue states during military training and physical exercise, the development of controllable and practical wearable fatigue biosensors must be the general trend.

## AUTHOR CONTRIBUTIONS

**Jingyang Zhang:** Conceptualization (equal); validation (equal); visualization (equal); writing – original draft (equal). **Mengmeng Chen:** Conceptualization (supporting); visualization (supporting). **Yuan Peng:** Formal analysis (supporting); investigation (supporting). **Shuang Li:** Conceptualization (supporting); investigation (supporting). **Dianpeng Han:** Supervision (supporting); visualization (supporting). **Shuyue Ren:** Investigation (supporting); visualization (supporting). **Kang Qin:** Formal analysis (supporting); investigation (supporting). **Sen Li:** Data curation (supporting); resources (supporting). **Tie Han:** Conceptualization (supporting); project administration (supporting); supervision (supporting). **Yu Wang:** Conceptualization (equal); supervision (equal); visualization (equal); writing – original draft (equal). **Zhixian Gao:** Funding acquisition (lead); project administration (lead); supervision (lead); writing – review and editing (lead).

## CONFLICT OF INTEREST

The authors declare no competing financial interest.

### PEER REVIEW

The peer review history for this article is available at https://publons.com/publon/10.1002/btm2.10318.

## Data Availability

The data used in this paper are all available.
